# *In vivo* and *ex vivo* cetuximab sensitivity assay using three-dimensional primary culture system to stratify *KRAS* mutant colorectal cancer

**DOI:** 10.1371/journal.pone.0174151

**Published:** 2017-03-16

**Authors:** Takahiro Tashiro, Hiroaki Okuyama, Hiroko Endo, Kenji Kawada, Yasuko Ashida, Masayuki Ohue, Yoshiharu Sakai, Masahiro Inoue

**Affiliations:** 1 Department of Biochemistry, Osaka Medical Center for Cancer and Cardiovascular Diseases, Osaka, Osaka, Japan; 2 Departmet of Surgery, Graduate School of Medicine, Kyoto University, Kyoto, Kyoto, Japan; 3 Charles River Laboratories Japan, Yokohama, Kanagawa, Japan; 4 Department of Surgery, Osaka Medical Center for Cancer and Cardiovascular Diseases, Osaka, Osaka, Japan; Baylor University Medical Center, UNITED STATES

## Abstract

In clinic, cetuximab, an anti-EGFR antibody, improves treatment outcomes in colorectal cancer (CRC). *KRAS*-mutant CRC is generally resistant to cetuximab, although difference of the sensitivity among *KRAS*-mutants has not been studied in detail. We previously developed the cancer tissue-originated spheroid (CTOS) method, a primary culture method for cancer cells. We applied CTOS method to investigate whether ex vivo cetuximab sensitivity assays reflect the difference in sensitivity in the xenografts. Firstly, in vivo cetuximab treatment was performed with xenografts derived from 10 CTOS lines (3 *KRAS*-wildtype and 7 *KRA*S mutants). All two CTOS lines which exhibited tumor regression were *KRAS*-wildtype, meanwhile all *KRAS*-mutant CTOS lines grew more than the initial size: were resistant to cetuximab according to the clinical evaluation criteria, although the sensitivity was quite diverse. We divided *KRAS*-mutants into two groups; partially responsive group in which cetuximab had a substantial growth inhibitory effect, and resistant group which exhibited no effect. The ex vivo signaling assay with EGF stimulation revealed that the partially responsive group, but not the resistant group, exhibited suppressed ERK phosphorylation ex vivo. Furthermore, two lines from the partially responsive group, but none of the lines in the resistant group, exhibited a combinatory effect of cetuximab and trametinib, a MEK inhibitor, ex vivo and in vivo. Taken together, the results indicate that ex vivo signaling assay reflects the difference in sensitivity in vivo and stratifies KRAS mutant CTOS lines by sensitivity. Therefore, coupling the in vivo and ex vivo assays with CTOS can be a useful platform for understanding the mechanism of diversity in drug sensitivity.

## Introduction

Colorectal cancer (CRC) is one of the leading causes of cancer prevalence and death worldwide. Treatment of CRC has progressed by the recent development of new drugs, such as oxaliplatin and irinotecan, as well as molecular target drugs, such as cetuximab, panitumumab, bevacizumab, aflibercept, regorafenib, and ramucirumab [1–3]. Anti-EGFR antibodies, cetuximab and panitumumab, have contributed remarkably to the treatment of metastatic CRC, though CRCs with *KRAS* mutations at codons 12 and 13 are resistant to these antibodies [4–6]. *NRAS* mutations and mutations at other codons in *KRAS* also reportedly contribute to cetuximab resistance [7–9]. Currently, expanded *RAS* wildtype CRCs (*KRAS* and *NRAS* wildtype) are candidates for anti-EGFR therapy [1]. To improve treatment outcomes for CRC, it is important to develop effective therapies for *RAS* mutants.

Established cancer cell lines have contributed to the study of cancer and development of new drugs, though discrepancies are often observed between experimental results and clinical trials, possibly due to changes or severe selection during the establishment and passage of cells in vitro [10, 11]. Inter-patient heterogeneity may not be maintained in established cell lines [12, 13]. On the other hand, patient-derived xenografts (PDXs) better preserve the original characteristics of patient tumors, and the assay results reflect the clinical trials [12–17]. However, the costly and time consuming assay of PDXs is not suitable for testing multiple candidate drugs or studying detailed signaling pathways.

Previously, we developed the cancer tissue-originated spheroid (CTOS) method, a preparation and culture method for primary cancer cells from patient tumors[18]. By maintaining cell-cell contact throughout the process, we can avoid anoikis and prepare pure cancer cells stably and efficiently. We previously reported the successful preparation of CTOSs from various cancers, including colon, lung, bladder, brain, and uterine cancer [18–23]. CTOSs preserve the original characteristics both ex vivo and in vivo [18–20, 22, 23]. CTOSs can also be prepared efficiently from CTOS-derived xenograft tumors and subjected to ex vivo experiments.

As CRC PDX models have been reported to reflect the results of clinical trials for cetuximab [14–17], we investigated whether an ex vivo cetuximab sensitivity assay using CTOSs can reflect the results of an in vivo study using CTOS xenografts. Using the ex vivo platform, we attempted to find biomarkers and the effective drugs to combine with cetuximab for KRAS mutant CRC.

## Materials and methods

### CTOS preparation, culture, and cryopreservation

The preparation of CTOSs from CRC patients was performed as described previously [18]. Briefly, surgical specimens were obtained from Osaka Medical Center for Cancer and Cardiovascular Diseases after obtaining informed consent. The surgical specimens were mechanically and enzymatically digested into small fragments. Materials retained by 100 μm or 40 μm cell strainers (BD Falcon, Franklin Lakes, NJ) were collected and cultured in suspension in StemPro hESC (Invitrogen, Carlsbad, CA) with 8 ng/ml of bFGF (Invitrogen) to form CTOSs. Frozen stocked CTOSs were thawed and xenograft tumors generated as described above. CTOSs were prepared from the xenografts and subjected to further analysis. Cryopreservation was performed using StemCell Keep (BioVerde, Kyoto, Japan).

### Animal studies

Animal studies were approved by the Institutional Animal Care and Use Committee of Osaka Medical Center for Cancer and Cardiovascular Diseases and performed in compliance with the institutional guidelines. For cetuximab mono-therapy, a mixture of CTOSs and Matrigel was transplanted into the flank of NOD/SCID mice (Charles River Laboratories Japan, Yokohama, Japan). When the tumor reached 160 mm^3^, cetuximab was injected intraperitoneally twice a week at 20 and 60 mg/kg. For the combination therapy, tumors were generated as described above using BALB/cAJcl-nu/nu mice (CLEA Japan, Tokyo, Japan). When the tumor reached 300 mm^3^, cetuximab was injected intraperitoneally twice a week at 20 mg/kg and trametinib administrated orally every day at 0.3 mg/kg. Trametinib was suspended in 0.5% methyl cellulose with 0.2% Tween80. Tumor size was measured twice a week and the tumor volume calculated as follows: 0.5 x width^2^ x length. For ethical reasons mice bearing an excessive tumor volume (>2,000 mm^3^) were euthanized.

### Grouping of CTOS lines by sensitivity to cetuximab in vivo

CTOS lines were classified into three groups according to their sensitivity to cetuximab. The regression group consisted of the lines in which the average tumor volume at day 11 was the same or less than the average starting volume. The partially responsive group consisted of lines in which the average tumor volume was suppressed more than 10% compared to the average volume of non-treated tumors but did not show regression. The resistant group consisted of lines in which the average tumor volume at day 11 was suppressed less than 10% compared to the average volume of non-treated tumors.

### Mutational analysis

Mutational analysis was performed using Ion AmpliSeq™ Cancer Hotspot Panel v2 (ThermoFisher, Waltham, MA) and next-generation sequencing (TAKARA, Kusatsu, Japan).

### Immunohistochemistry

Formalin-fixed paraffin-embedded samples were used for immunohistochemistry as described previously [18]. Antigen retrieval was performed by autoclave incubation in citrate buffer (pH 6.0). Primary antibody specific for the EGFR (clone D38B1) was obtained from Cell Signaling Technologies (Danvers, MA). Images were acquired using the CellSens standard imaging software (Olympus, Tokyo, Japan). Hematoxylin was used for counter staining. The staining intensity was evaluated as low if membranous staining was present in less than 10% of tumor cells, med if membranous staining was present in 10% to 50% of tumor cells, and high if membranous staining was present in more than 50% of tumor cells.

### Reagents

Trametinib was purchased from Selleck Chemicals (Houston, TX, USA). Drug screening was performed using SCADS Inhibitor Kit IV. The drugs were dissolved in DMSO and used below 0.1% DMSO.

### CTOS-based sensitivity assay

After CTOS preparation, the CTOSs were cultured in suspension overnight in the standard CTOS medium (StemPro hESC plus bFGF). Each CTOS was embedded in a gel droplet of Matrigel GFR (BD Biosciences, Bedford, MA) and cultured for 7 days in the standard CTOS medium or DMEM containing 10% fetal bovine serum (FBS medium) containing the indicated doses of cetuximab. In the case of neuregulin 1 (NRG1) / heregulin (HRG) stimulation, 10 ng/ml of HRG (Peprotech, Rocky Hill, NJ) was added to the FBS medium. The effect was evaluated by comparing the area of the spheroid to that of day 0 as measured using Image J software. (National Institutes of Health, Bethesda, MD).

### Reconstituted spheroid-based sensitivity assay

After washing the CTOSs with PBS, they were dissociated into single cells using 0.25% trypsin/EDTA and filtered through a 40 μm cell strainer (BD Falcon). Approximately 1x10^4^ cells/100ul were seeded in poly-HEMA-coated 96-well plates and cultured in the FBS medium and 10 μM of ROCK inhibitor Y27632 (Wako, Osaka, Japan). Each drug was added and the CTOSs cultured for 7 days. ATP content was measured at day 7 by CellTiter-Glo Luminescent Cell Viability Assays (Promega, Madison, WI, USA) and adjusted to the content of the vehicle-treated control.

### Western blot

For signaling assays using CTOSs, the medium was changed to the basal medium (DMEM/F12 containing 1.8% BSA, 1x nonessential amino acids, 100 U/ml penicillin, 100 μg/ml streptomycin [all from Invitrogen], 0.1 mM β-mercaptoethanol [Wako]), the standard CTOS medium, or the FBS medium the day after CTOS preparation. The CTOSs were cultured overnight, treated with 100 nM cetuximab for 2 hours, and the samples collected. For signaling assays using reconstituted spheroids, the spheroids were treated with the drugs 2 days after re-aggregation and samples collected 2 hours after treatment. In the case of EGF stimulation, 10 ng/ml of EGF (Invitrogen) was added 15 min before sample collection. Immunoblots were performed as described previously [18]. Primary antibodies against EGFR (clone D38B1), pEGFR (Tyr1068) (clone D7A5), pHER2 (Y1221/1222) (clone 6B12), pHER3 (Tyr1289) (clone 21D3), AKT (clone 40D4), pAKT (Ser473) (clone D9E), p44/42 MAPK (clone 3A7), and pp44/42 MAPK (Thr202/Tyr204) (clone D13.14.4E) were obtained from Cell Signaling Technologies, and anti β-actin (clone AC-15) from Sigma-Aldrich (St. Louis, MO).

### Statistical analysis

One-way or two-way ANOVA followed by Bonferroni’s post-test was used for comparisons of multiple groups, and correlations were analyzed by Pearson’s correlation using GraphPad Prism 6 software (San Diego, CA). P<0.05 was considered significant.

## Results

### In vivo cetuximab sensitivity assay using CTOS lines stratified *KRAS* mutant colorectal cancer

**Fig 1:**
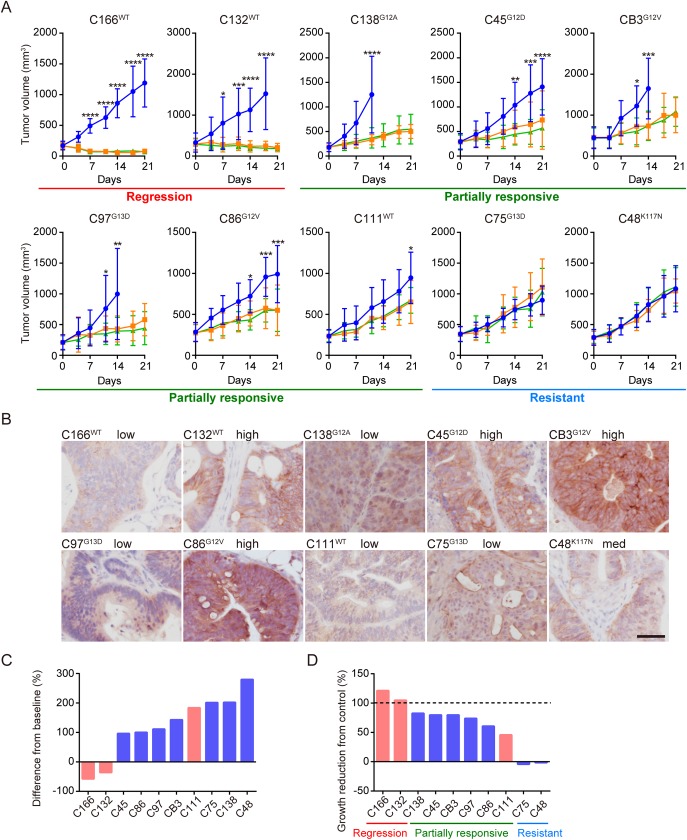
In vivo cetuximab sensitivity assay revealed diverse effect on 10 CTOS-derived xenogfrafts. A, Growth curves of subcutaneous tumors originating from colorectal CTOS lines. Blue, treated with vehicle; orange, cetuximab (20 mg/kg); green, cetuximab (60 mg/kg). Mean±SD is shown. N = 4–6 in each treated group. *P < 0.05, **P < 0.01, ***P < 0.001, ****P < 0.0001; 60 mg/kg cetuximab versus control; two-way ANOVA with Bonferroni post-test. Regression, partially responsive, and resistant are explained in the text. The type of *KRAS* mutant is indicated in superscript to the left of the line name. B, Microscopic images of vehicle-treated xenografts in A stained with EGFR antibody. Scale bar, 50 μm. Grading by staining intensity is shown. C, Waterfall plot of cetuximab-treated tumor growth (differences from baseline). The average sizes of cetuximab (60 mg/kg) treated tumors at day 21 were subtracted by the sizes at day 0 and the ratio compared to the average sizes at day 0 as follows: (V_Cmab_ (day 21)—V_Cmab_ (day 0))/ V_Cmab_ (day 0) x 100. Red bars, wildtype *KRAS* tumors; blue, *KRAS* mutants. D, Growth reduction from control (vehicle treated) tumors. The average sizes of vehicle-treated tumors at day 11 were subtracted by the sizes of tumors treated with cetuximab (60 mg/kg) and the ratio compared to the average sizes at day 11 as follows: (V_vehicle_(day 11)- V_Cmab_(day 11))/ V_vehicle_(day 11) x 100.

We established 10 CTOS lines from patients with CRC (Table 1). Among these 10 lines, three were *KRAS*-wildtype and seven were *KRAS* mutants. CTOSs were injected subcutaneously into NOD-SCID mice to create CTOS-derived xenografts, which were subjected to the cetuximab sensitivity assay in vivo. The sensitivity varied among the lines, some of which exhibited tumor regression (regression group), a partial response (partially responsive group), or resistance (resistant group) (Fig 1A). EGFR staining did not correlate with cetuximab sensitivity in the xenograft tumors (Fig 1B) as previously reported for clinical samples [24]. The clinically used waterfall plot, in which tumor volume is compared to the volume at the starting point, is shown in Fig 1C. Both lines in the regression group were *KRAS*-wildtype, whereas all of the *KRAS* mutants grew more than the initial volume and were in the partially responsive or resistant groups. The results were compatible with previous clinical reports and studies using PDX mouse models [5, 15–17].

**Table 1:** Clinical and genomic profiles of 10 CTOS lines.

	Clinical Information				Mutational status of CTOS						
Sample ID	Patient sex	Tumor location	Tumor histology	TNM stage	*KRAS*	*NRAS*	*BRAF*	*PIK3CA*	*AKT1*	*APC*	*TP53*
C166	F	Ra	Mod	ⅢB	WT	WT	WT	WT	WT	WT	WT
C132	F	T	Mod	ⅢB	WT	WT	WT	WT	WT	MT	WT
C111	F	Rs	Mod	ⅣB	WT	WT	WT	WT	WT	MT	MT
C138	F	A	Mod	ⅡA	G12A	WT	WT	WT	WT	MT	WT
C45	M	Ra	Mod	ⅣA	G12D	WT	WT	WT	WT	MT	WT
CB3	F	A	Well	ⅡA	G12V	WT	WT	WT	WT	MT	WT
C97	F	Rb	Mod	ⅢC	G13D	WT	WT	MT	WT	WT	MT
C86	M	S	Mod	ⅣA	G12V	WT	WT	WT	WT	MT	MT
C75	F	Ra	Mod	ⅣA	G13D	WT	WT	WT	MT	MT	WT
C48	M	Rab	Mod	ⅡA	K117N	WT	WT	MT	WT	MT	WT

Patient sex: M, male; F, female. Tumor location: A, ascending colon; T, transverse colon; S, sigmoid colon; Rs, rectosigmoid; Ra, upper rectum; Rb, lower rectum. Tumor histology: Well, well differentiated adenocarcinoma, Mod, moderately differentiated adenocarcinoma. Mutational status of CTOS: A panel of 50 frequently mutated genes in cancer was annalyzed. No mutations other than those mentioned above were detected. WT, wild type, MT, mutant. Mutations are shaded gray.

Experimental settings allowed growth reduction to be assessed from control (vehicle treated) tumors (Fig 1D). The difference between the partially responsive group and resistant group was clear. Thus, *KRAS* mutant CTOS lines were divided into two groups by cetuximab sensitivity: the partially responsive group and the resistant group. We assessed hotspot mutations of 50 genes. Neither *KRAS* mutation type nor any single mutation correlated with the difference (Table 1).

### Optimizing conditions for ex vivo cetuximab sensitivity assay for CTOSs

**Fig 2:**
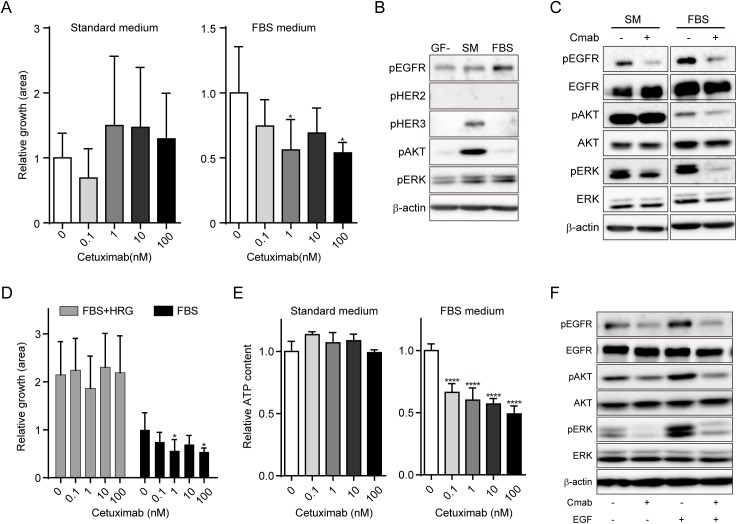
Establishment of ex vivo cetuximab sensitivity assays. A, Growth of C132 CTOSs cultured with the indicated doses of cetuximab for 7 days. The relative growth at day 7 adjusted by day 0 is shown. Culture media are indicated. Mean±SD is shown. N = 3–6. Significance of the decrease in relative growth. *P < 0.05, versus 0; one-way ANOVA with Bonferroni post-test. B, Western blots of lysates from C132 CTOSs cultured in the indicated media for 24 h. GF-, basal medium without any growth factor; SM, the standard CTOS medium; FBS, the FBS medium. Each antibody is indicated. C, Western blots of lysates from C132 CTOSs treated with or without cetuximab (100 nM) for 2 h. CTOSs were cultured in the indicated media for 24 h before cetuximab treatment. Cmab, cetuximab. D, Growth of C132 CTOSs cultured with the indicated doses of cetuximab for 7 days. CTOSs were cultured in the FBS medium with (gray bars) or without (black bars, same as Fig 2A) 10 ng/ml HRG. The relative growth at day 7 adjusted by day 0 is shown. Mean±SD is shown. N = 5–6. Significance of the decrease in relative growth. *P < 0.05, versus 0; one-way ANOVA with Bonferroni post-test. E, Results of the reconstituted spheroid-based assay. The viable cell number in the spheroids was evaluated by ATP content, and the ratio to that of non-treated spheroids is shown. CTOSs were cultured for 7 days in the indicated media with the indicated doses of cetuximab. Mean±SD is shown. N = 5–6. Significance of the decrease in relative ATP content. ****P < 0.0001, versus 0; one-way ANOVA with Bonferroni post-test. F, Western blot evaluation of intracellular signaling ex vivo. Reconstituted spheroids generated from C132 CTOSs were treated with or without 100 nM cetuximab for 2 h and then stimulated with or without 10 ng/ml of EGF for 15 min.

To investigate whether in vivo and ex vivo cetuximab sensitivity assays correlate, we tested the C132 CTOS line from the in vivo regression group. CTOS growth was not suppressed by cetuximab in the standard CTOS medium (Fig 2A). The media contained HRG [19], a ligand of HER3, the activation of which has been reported to be one of the mechanisms of cetuximab resistance [15, 25]. Alternatively, we examined another media containing 10% fetal bovine serum (FBS medium), and found that C132 CTOSs were sensitive to cetuximab (Fig 2A). The basal status of intracellular signaling revealed a remarkable difference between the media. In the standard CTOS medium, basal levels of HER3 and AKT phosphorylation were higher (Fig 2B), and AKT and ERK phosphorylation was suppressed less by cetuximab than in the FBS medium (Fig 2C). Adding HRG to the FBS medium abolished the growth inhibitory effect of cetuximab (Fig 2D). Thus, HER3 activation by HRG in the standard CTOS medium obscured the effect of cetuximab ex vivo. In addition, to reduce the deviation in growth among CTOSs (Fig 2A), we dissociated C132 CTOSs into single cells and reconstituted the spheroids by culturing the cells at high density in a low attachment culture dish. The decrease in deviation was prominent in the assay using reconstituted spheroids compared to CTOSs (Fig 2A and 2E), and the relative ATP levels were confirmed to be remarkably decreased by cetuximab treatment in the FBS medium but not the standard CTOS medium (Fig 2E). Using the reconstituted spheroids, phosphorylation of EGFR, AKT, and ERK was suppressed by cetuximab treatment with or without EGF stimulation (Fig 2F). Therefore, we applied the reconstituted spheroids in FBS medium in further evaluations.

### Suppression of ERK phosphorylation ex vivo paralleled *KRAS* mutant cetuximab sensitivity in vivo

**Fig 3:**
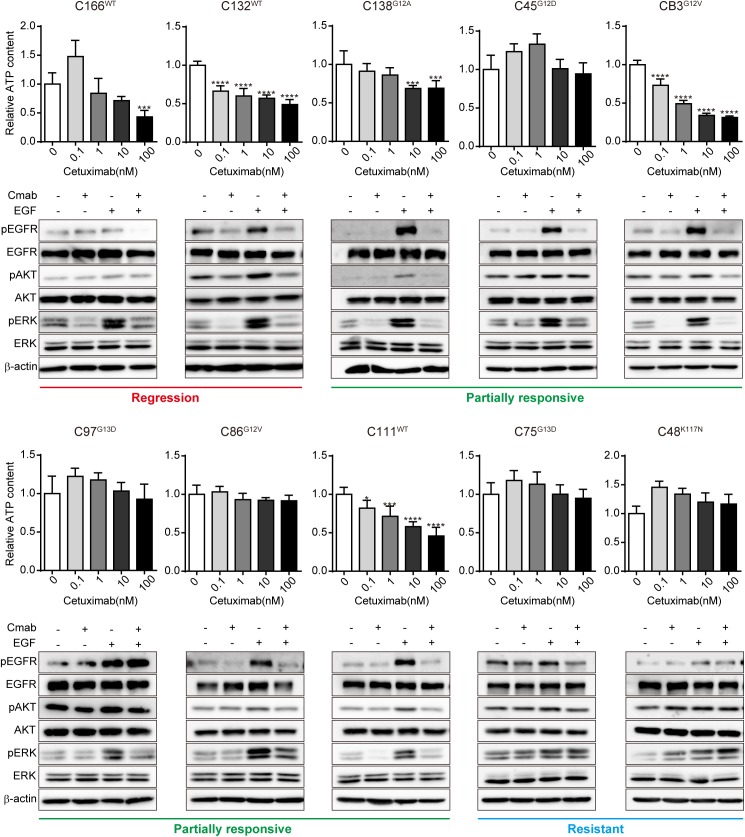
Response of ERK phosphorylation to cetuximab treatment stratified *KRAS* mutants into two groups ex vivo. The result of the reconstituted spheroid-based assay in each CTOS line is shown. The CTOS lines are ordered according to the results of the in vivo assay and the grouping indicated below. The type of *KRAS* mutant is indicated in superscript to the left of the line name. Upper panels: relative ATP content of reconstituted spheroids cultured with the indicated doses of cetuximab for 7 days. Mean±SD is shown. N = 3–6. Significance of the decrease in relative ATP content. *P < 0.05, ***P < 0.001, ****P < 0.0001, versus 0; one-way ANOVA with Bonferroni post-test. Lower panels: ex vivo signaling assay by Western blot. Reconstituted spheroids were treated with or without 100 nM cetuximab for 2 h and then stimulated with or without 10 ng/ml EGF for 15 min. Cmab, cetuximab.

We performed ex vivo growth assays and signaling assays using reconstituted spheroids (Fig 3). Substantial differences were observed among the lines. The growth suppression by cetuximab in vivo was correlated to the ex vivo growth, as well as ERK phosphorylation with or without EGF stimulation (S1 Fig).

The suppression of growth and ERK phosphorylation without EGF stimulation was distinct between the regression and resistant groups (2/2 vs. 0/2), whereas the responses varied among the partially responsive group. For example, C111 exhibited markedly discrepant results between in vivo and ex vivo assays. In contrast, with EGF stimulation, ERK phosphorylation was suppressed not only in the regression group, but also in the partially responsive group. It was still not suppressed in the resistant group. The results indicate that the resistant group has quite distinct characteristics that can be better distinguished by sustained ERK phosphorylation after cetuximab treatment in the presence of EGF stimulation ex vivo. In regards to the mutational status of *KRAS*, the seven *KRAS* mutant lines were distributed into two groups: partially responsive (5/7) and resistant (2/7). Thus the *KRAS* mutants were stratified into two groups based on the effect of cetuximab not only in vivo but also ex vivo.

### Combination of cetuximab and trametinib was effective in the partially responsive group ex vivo

**Fig 4:**
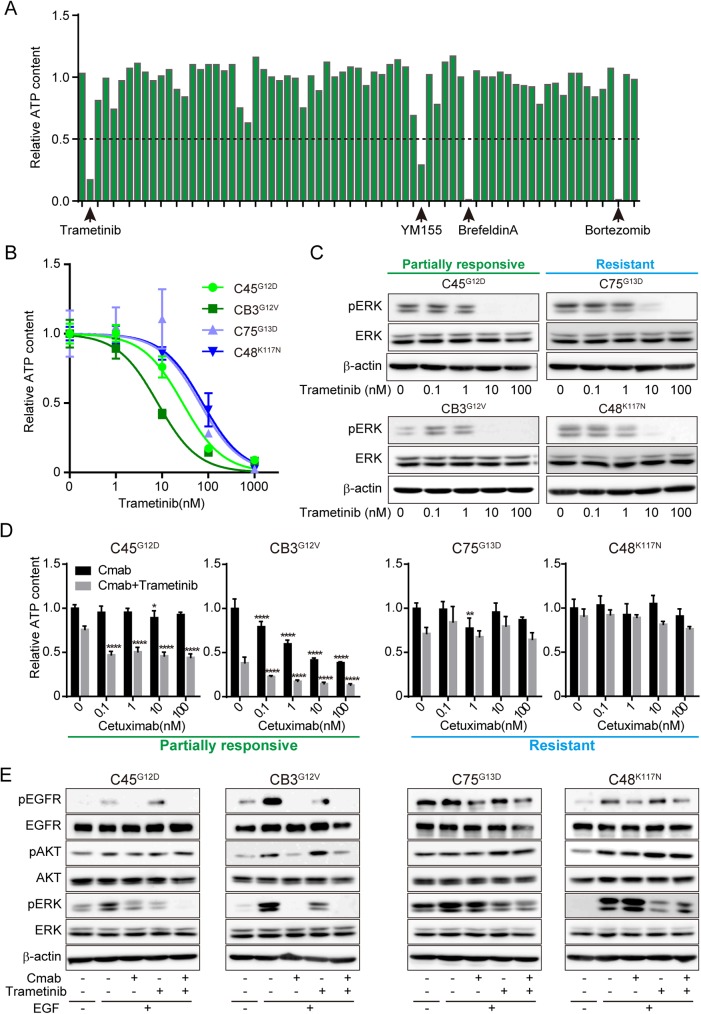
Combination of cetuximab and trametinib was more effective in the partially responsive group ex vivo. A, Screening of 71 drugs using reconstituted C45 spheroids. The spheroids were cultured with 100 nM of drug for 7 days. The drugs for which the relative ATP content was less than 0.5 are indicated. Mean values are shown. N = 4. B, Dose-response curve of trametinib in four CTOS lines (the partially responsive group, C45 and CB3; the resistant group, C75 and C48). The type of *KRAS* mutant is indicated in superscript to the left of the line name. C, Lysates of reconstituted spheroids from four CTOS lines were subjected to Western blotting. The spheroids were treated with the indicated doses of trametinib for 4 h. The antibodies used are indicated. D, Relative ATP content of reconstituted spheroids cultured for 7 days with cetuximab alone at the indicated doses (black bars), or in combination with 10 nM of trametinib (gray bars). Mean±SD is shown. N = 6. Significance of the decrease in relative ATP content. *P < 0.05, **P < 0.01, ****P < 0.0001, versus 0; one-way ANOVA with Bonferroni post-test. E, Western blots of lysates from the reconstituted spheroids treated with or without 100 nM cetuximab, 5 nM trametinib, or a combination of 100 nM cetuximab and 5 nM trametinib for 2 h with or without stimulation with 10 ng/ml EGF for 15 min.

As the *KRAS* mutant CRC CTOS lines in the partially responsive group exhibited a partial response while those in the resistant group exhibited no response, we expected that combination therapy may improve the effect of cetuximab in the partially responsive group. First, we screened 71 signaling inhibitors as single reagents using reconstituted spheroids derived from C45 CTOSs, a line from the partially responsive group (Fig 4A and S1 Table). Four drugs resulted in growth inhibition in which the relative ATP content was <0.5. One drug was trametinib, a MEK inhibitor. As MEK is a downstream molecule of *KRAS*, we further studied trametinib.

To determine the dose of trametinib for the combination treatment assays, we examined mono-treatment with trametinib at various doses (Fig 4B and 4C). The C75 and C48 lines in the resistant group were relatively resistant in the growth assay, although ERK phosphorylation was remarkably inhibited in all lines examined when the doses were more than 10 nM (Fig 4B and 4C). Therefore, we chose 10 nM for the growth assay (Fig 4D). Combination treatment in the partially responsive group (C45, CB3) effected growth to a greater extent than trametinib mono-treatment, but this was not observed in the resistant group (C75, C48) (Fig 4D). For the signaling assay, we chose 5 nM trametinib with EGF treatment to ensure a clear combinatory effect (Fig 4E). In the partially responsive group (C45, CB3), ERK phosphorylation was suppressed more by combination treatment than trametinib alone, with (Fig 4E) or without (S2 Fig) EGF stimulation. In contrast, combination treatment did not suppress ERK phosphorylation in the resistant group (C75, C48).

### Combination of cetuximab and trametinib was more effective in the partially responsive group than in the resistant group in vivo

**Fig 5:**
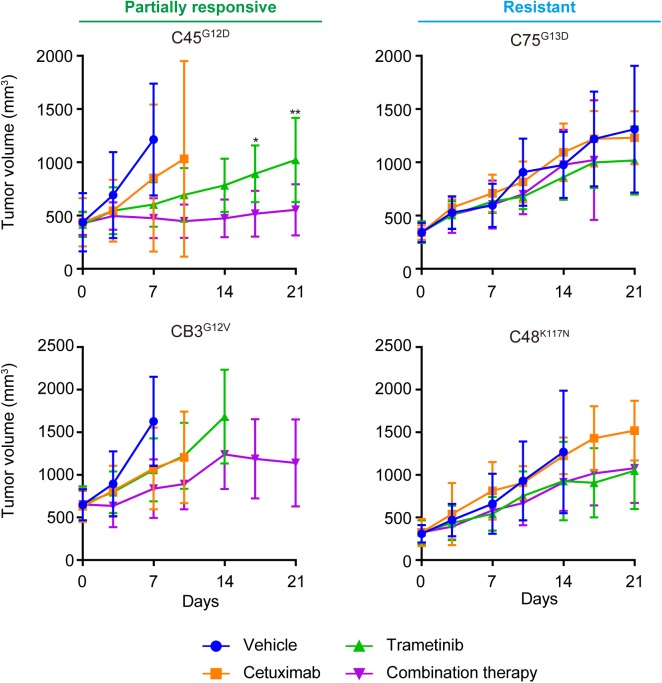
Combination of cetuximab and trametinib was more effective in the partially responsive group in vivo. Growth curves of subcutaneous tumors generated by four CTOS lines (the partially responsive group, C45 and CB3; the resistant group, C75 and C48). Blue, treated with vehicle; orange, cetuximab (20 mg/kg) alone; green, trametinib (0.3 mg/kg) alone; purple, combination of cetuximab (20 mg/kg) and trametinib (0.3 mg/kg). Mean±SD is shown. N = 6 in each treated group. *P < 0.05, **P < 0.01, mono therapy with trametinib versus combination; two-way ANOVA with Bonferroni post-test. The type of *KRAS* mutant is indicated in superscript to the left of the line name.

We examined the combined effect of cetuximab and trametinib in vivo (Fig 5) using xenograft models generated by subcutaneously injecting the CTOSs. Mono-therapy with either cetuximab or trametinib had significant effects in the partially responsive group (C45, CB3), and the combination of cetuximab and trametinib enhanced the effect; CB3 tumors stopped growing after 14 days with combination therapy. In contrast, the lines in the resistant group (C75, C48) exhibited a marginal effect with trametinib alone, and combination therapy seemingly had no effect.

## Discussion

We applied the CTOS method in this study of in vivo and ex vivo drug sensitivity assays. The in vivo results were consistent with previous reports of the cetuximab resistance in *KRAS* mutant CRCs. Here we revealed that *KRAS* mutant CRC tumors have a wide range of sensitivity to cetuximab and can be divided into two groups: partially responsive and resistant. Lines in the partially responsive group exhibit remarkable suppression of ERK phosphorylation after cetuximab treatment in the presence of EGF stimulation. Combination therapy with cetuximab and trametinib, a MEK inhibitor, was more effective than mono-treatment in the partially responsive group but not the resistant group.

Recently, the primary culture of cancer cells has progressed remarkably and is expected to be applied to the prediction of sensitivity for individual patients. A phenotypic assay can be the basis for studying mechanisms and developing biomarkers for a drug, especially when multiple factors are involved [26]. CTOSs can be prepared efficiently from patient tumors, as well as PDX or CTOS-derived xenografts. In addition, CTOSs can be proficiently cryopreserved as spheroids. We previously reported that the ex vivo growth assay for selected CTOSs correlates with the in vivo assay for various cancers and drugs[19, 22, 23, 27]. To the best of our knowledge, this is the first report to perform and compare in vivo and ex vivo cetuximab sensitivity assays side by side for a panel of CRC primary cultures. The results of the in vivo assays using CTOS-derived xenografts were compatible with previous reports using PDX models. Furthermore, the ex vivo signaling assay reflected the in vivo sensitivity, although the ex vivo growth did not completely reflect it.

The CTOS line C132 was sensitive to cetuximab in vivo but resistant ex vivo when the standard CTOS medium containing HER3 ligand was used. As the growth conditions in culture can never be exactly the same as the microenvironment in vivo, the culture conditions need to be adjusted to conform the results of the ex vivo growth assay to those of the in vivo assay or patient responses. Various factors, such as the selection of growth factors and cell matrices and co-culture with fibroblasts, should be optimized. On the other hand, signaling assays are more promising because they assess the ‘potential capacity’ of cancer cells without long-term culture. In this study, the suppression of ERK phosphorylation under high-dose EGF-stimulated conditions was the best predictor of cetuximab sensitivity in vivo, although the functional role of suppressing ERK phosphorylation is still not clear.

*KRAS* mutant CRCs are thought to be generally resistant to cetuximab, as the overall survival, progression-free survival, and response rate have not improved in clinical trials [5, 6]. All *KRAS* mutant xenograft tumors in this study grew after cetuximab treatment, which is consistent with findings from previous clinical and pre-clinical studies [5, 14–17]. However, by comparing the tumors to vehicle-treated controls, which is only allowed in experimental settings, we demonstrated that *KRAS* mutant CRCs can be divided into two groups. Tumor growth was significantly suppressed by cetuximab treatment in the partially responsive group but not the resistant group. In previous clinical reports, 10% of *KRAS* mutant CRC exhibited disease control [28], and mutations in codon 13 (G13D) have been suggested to be more sensitive to cetuximab than mutations in codon 12 [29, 30]. Thus, diverse sensitivities to cetuximab, even in *KRAS* mutants, have been suggested in clinical studies, although the characteristics and mechanisms are poorly understood. When the previous reports using PDX are examined carefully, they also indicate that *KRAS* mutants exhibit various levels of partial response to cetuximab [16, 17]. Thus, a partial response to cetuximab in *KRAS* mutant CRC was observed in both CTOS xenografts and PDX models, although the clinical relevance remains to be clarified. In this study, attributing the different sensitivities to the type of *KRAS* mutation is not likely, as all four of the *KRAS* mutations in codon 12 were in the relatively sensitive (partially responsive) group, and two of the G13D (C97 and C75) lines were in different sensitivity groups. Neither hotspot mutation in *NRAS*, *BRAF*, *PIK3CA* nor that of other genes which were reportedly related to cetuximab resistance [15, 31–33] was able to distinguish the two groups. Triad mutations of *APC*, *KRAS*, and genes in PI3K-AKT pathway were found only in the resistant group. Since the number of the resistant lines are too small, further study should be performed to ascertain whether the triad mutations can be a biomarker of the resistant group.

In this study, the CTOS method enabled ex vivo signaling assays of cancer cells in xenograft tumors. Because *KRAS* is a downstream molecule in EGFR signaling, *KRAS* mutant CRCs are thought to be resistant to cetuximab due to constitutive activation of downstream signaling [34–36]. In this study, increased ERK phosphorylation was suppressed by cetuximab in all of the CTOS lines in the partially responsive group, indicating that ERK phosphorylation is still dependent on upstream signaling in the majority of *KRAS* mutant lines. The results are a sharp contrast to most previous reports using established CRC cell lines, in which ERK phosphorylation is mostly resistant to cetuximab in *KRAS* mutants [36–39]. The discrepancy may be attributed to the different culture conditions, or the partially responsive group may be rare in the established cell lines. On the other hand, the two lines in the resistant group did not respond to cetuximab treatment in regards to both growth and ERK phosphorylation. Particularly in C48 CTOSs, ERK phosphorylation increased with cetuximab treatment. The activation of compensatory pathways or relief of a negative feedback loop [40] may be involved.

Encouraged by the partial response to cetuximab in the partially responsive group, we investigated combination therapy with a MEK inhibitor. MEK is a downstream molecule of EGFR and RAS signaling, and MEK inhibitor is expected to enhance the effect of cetuximab [36, 38]. In this study, ERK phosphorylation and the growth of CTOSs were suppressed in two *KRAS* mutants in the partially responsive group after combination treatment. In contrast, the growth of CTOSs in two *KRAS* mutants in the resistant group were not affected by combination treatment. As for ERK phosphorylation, cetuximab had no additional effect on MEK inhibition (Fig 4E). The activation of pathway molecules between EGFR and ERK may be independent of EGFR activation. Alternatively, pathways other than MEK-ERK can be involved in cetuximab resistance, as MEK inhibition had a minor effect on growth (Fig 4B and 4C).

Previous studies offer some examples in which the combination of cetuximab and a MEK inhibitor is effective, even in resistant-type cell lines [36, 38]. The discrepancy may be due to the difference between CTOSs and established cell lines, or a difference in the MEK inhibitors used [37]. Resistant CTOS lines need to be accumulated to confirm the characteristics and investigate the mechanism.

Taken together, our data support CTOSs of the same origin as a model shuttling system between in vivo and ex vivo assays, especially for assessing the diversity of drug sensitivity. In addition, this approach may be applicable to existing PDX models, although further studies are required to clarify the similarity and difference between CTOS-derived xenograft and PDX model. Furthermore, ex vivo assays using CTOS technology may be useful for screening drugs and combination treatments or selecting patients for the therapies.

## Supporting information

S1 Fig Scatter plots and regression lines showing the correlation between in vivo growth reduction and various factors. Pearson’s correlation coefficients, R, and p-values are shown. In vivo growth reduction was the average rate of growth reduction day 11 after the first treatment with 60 mg/kg cetuximab in vivo. Ex vivo growth reduction was the average rate of growth reduction day 7 after treatment with 100 nM cetuximab ex vivo. The reduced intensity of ERK/AKT phosphorylation, which was adjusted by β-actin, was detected by Western blotting with (EGF+) or without (EGF-) stimulation ex vivo.
(TIF)

S2 Fig Ex vivo signaling assay of combination therapy with cetuximab and trametinib without EGF stimulation. Western blotting of lysates from the reconstituted spheroids treated with or without 100 nM cetuximab, 1 nM trametinib, or a combination of 100 nM cetuximab and 1 nM trametinib for 2 h without EGF stimulation. The type of *KRAS* mutant is indicated in superscript to the left of the line name.
(TIF)

S1 Table List and effect of the drugs used for screening. ATP content relative to that of non-treated spheroids is shown. Effective results (<0.50) are indicated in pink.
(PDF)
