# Supplementary figures and images for: In vivo and ex vivo cetuximab sensitivity assay using three-dimensional primary culture system to stratify KRAS mutant colorectal cancer
Source: PLoS One. 2017 Mar 16;12(3):e0174151. doi: 10.1371/journal.pone.0174151 (PMC5354432; doi:10.1371/journal.pone.0174151)

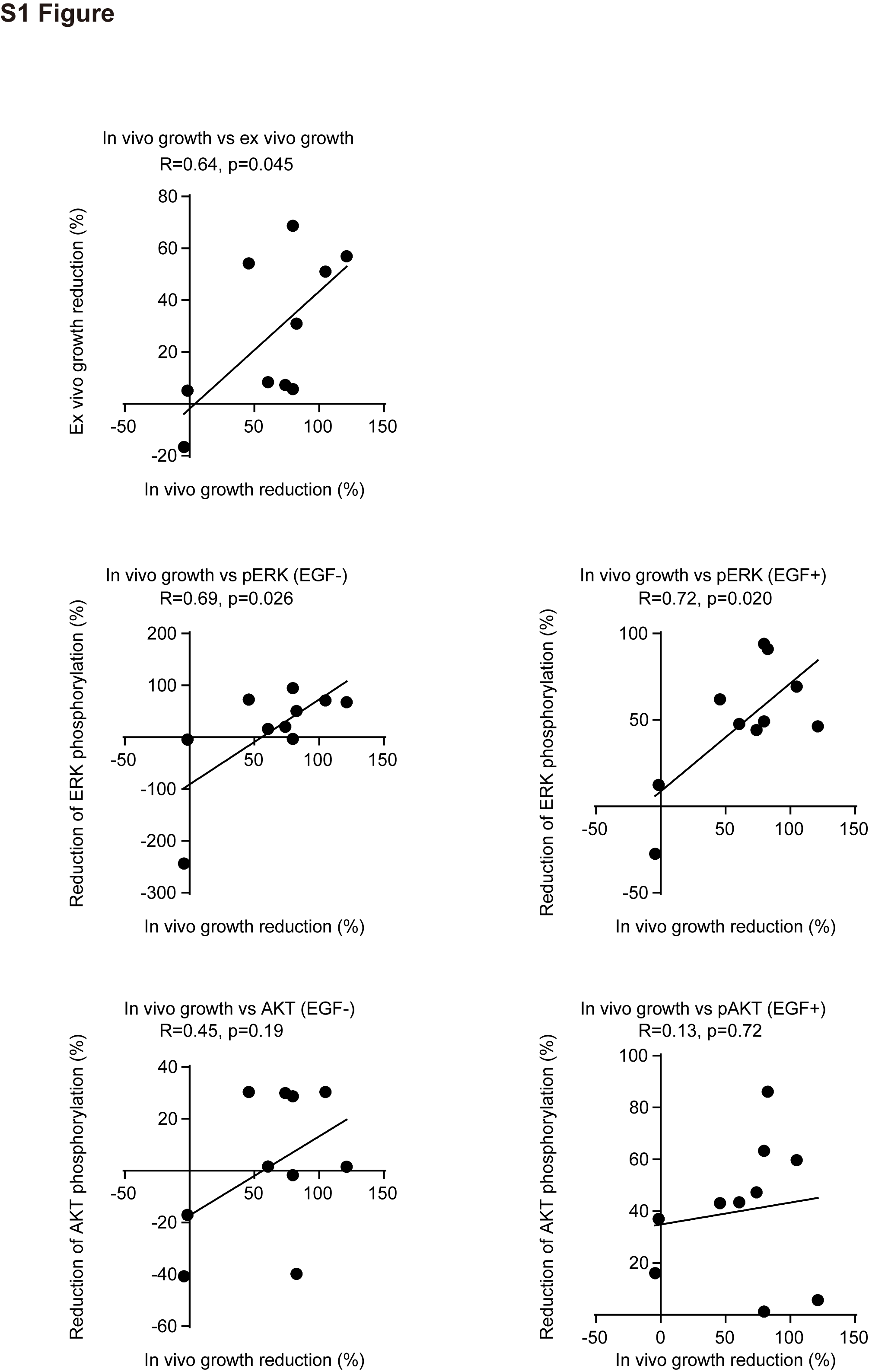

Supplement: S1 Fig — Pearson’s correlation coefficients, R, and p-values are shown. In vivo growth reduction was the average rate of growth reduction day 11 after the first treatment with 60 mg/kg cetuximab in vivo. Ex vivo growth reduction was the average rate of growth reduction day 7 after treatment with 100 nM cetuximab ex vivo. The reduced intensity of ERK/AKT phosphorylation, which was adjusted by β-actin, was detected by Western blotting with (EGF+) or without (EGF-) stimulation ex vivo. (TIF) [file pone.0174151.s001.tif]

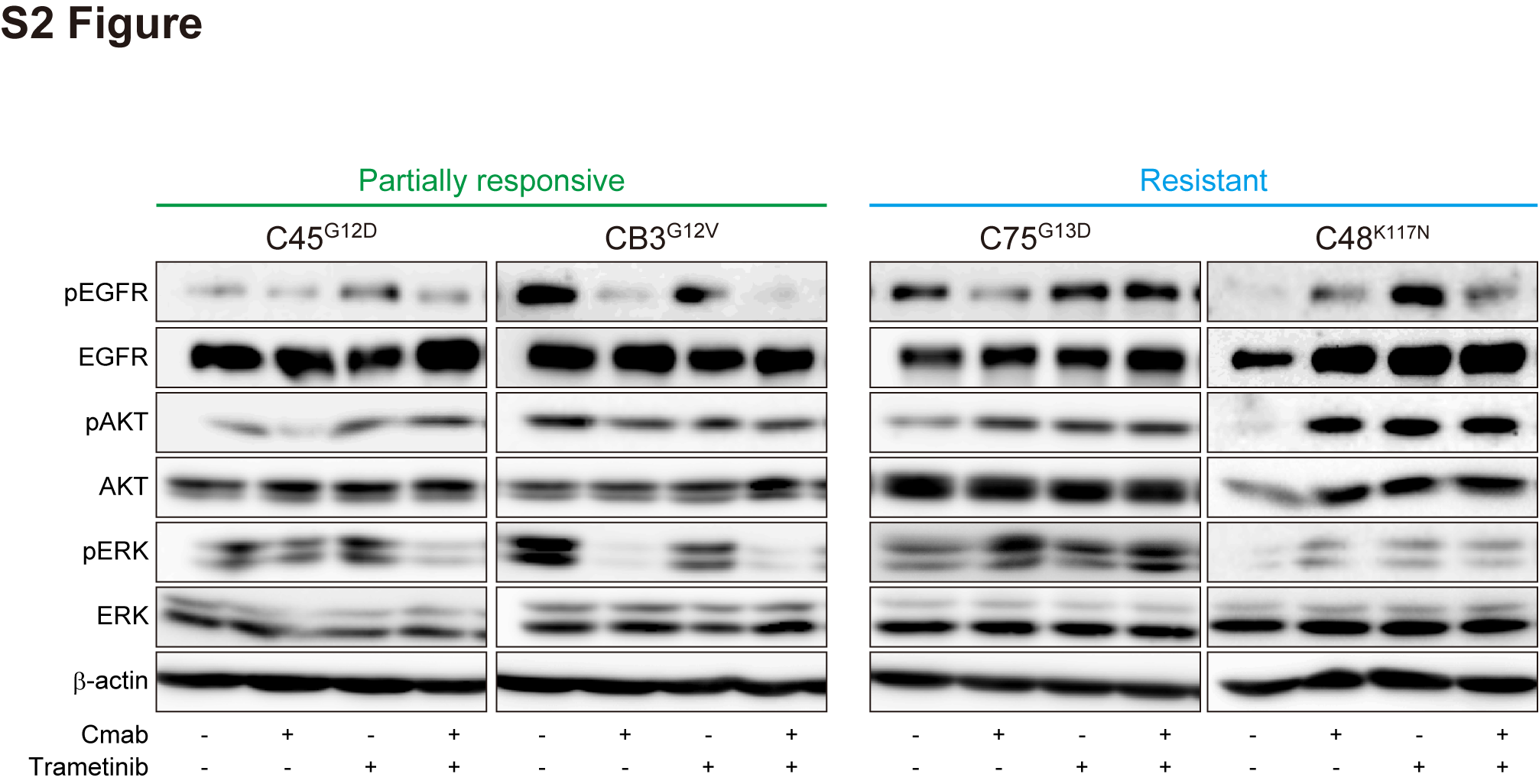

Supplement: S2 Fig — Western blotting of lysates from the reconstituted spheroids treated with or without 100 nM cetuximab, 1 nM trametinib, or a combination of 100 nM cetuximab and 1 nM trametinib for 2 h without EGF stimulation. The type of KRAS mutant is indicated in superscript to the left of the line name. (TIF) [file pone.0174151.s002.tif]
