# Supplementary material for: In vivo and ex vivo cetuximab sensitivity assay using three-dimensional primary culture system to stratify KRAS mutant colorectal cancer
Source: PLoS One. 2017 Mar 16;12(3):e0174151. doi: 10.1371/journal.pone.0174151 (PMC5354432; doi:10.1371/journal.pone.0174151)
Supplement: S1 Table — ATP content relative to that of non-treated spheroids is shown. Effective results (<0.50) are indicated in pink. (PDF) [file pone.0174151.s003.pdf]

S1 Table

List and effect of the drugs used for screening

Category	Inhibitor name	Relative ATP content
BRAF	Vemurafenib	1.03
MEK	trametinib	0.17
mTOR	temsirolimus	0.81
mTOR	Torkinib	0.99
mTOR	everolimus	0.74
EGFR	gefitinib	0.97
EGFR	erlotinib	1.07
EGFR/Her2	lapatinib	1.11
c-Met	PF-04217903	1.04
EML4-ALK	crizotinib	0.97
FGFR	PD173074	1.01
IGF-IR	OSI-906	1.06
TGFb-R	LY2157299	0.90
TGFb-R	SD208	0.84
Hedgehog	AY 9944	1.10
Hedgehog	cyclopamine	1.06
Hedgehog	Jervine	1.10
Hedgehog	Vismodegib	1.10
STAT3	WP1066	1.05
STAT3	5,15-DPP	1.10
PARP	BSI-201 (Iniparib)	0.75
PARP	ABT-888 (Veliparib)	0.63
PARP	MK-4827 (Niraparib)	1.16
pan-PARP	PJ-34	1.06
PARP-1/2-selective	Olaparib	1.00
tankyrase-selective PARP	XAV939	0.97
Wnt	IWP-2	1.01
Wnt	IWR-1-endo	0.99
GSK-3	BIO	0.75
GSK-3	TWS119	1.01
GSK-3	CT99021	0.89
Notch	DAPT	1.12
ALK	LDN193189	1.00
Bcr-Abl	nilotinib	1.04
Bcr-Abl/Kit	imatinib mesylate	1.08
Bcr-Abl/Src	dasatinib	1.07
Multi-kinases	sorafenib	0.93
Multi-kinases	sunitinib malate	1.04
Multi-kinases	pazopanib	1.02
Multi-kinases	Vandetanib	1.10
Multi-kinases	Axitinib	1.14
Aurora	ENMD-2076	1.08
Aurora	MLN8237	0.69
Survivin	YM155	0.29
Rho/SRF	CCG-1423	1.02
ROCK	Thiazovivin	0.78
PDK1	OSU-03012	1.12
JAK	Ruxolitinib	1.17
PIM	PIM1/2 Kinase Inhibitor V	1.00
golgi inhibitor	brefeldin A	0.01
thalidomide family	thalidomide	1.05
thalidomide family	lenalidomide	1.00
retinoids	tretinoin	1.00
retinoids	tamibarotene	1.04
DNA alkylation	temozolomide	1.02
AR	MDV3100	0.94
caspase activator	PAC-1	0.93
blc-2	ABT-737	0.92
G9a	UNC0638	0.78
G9a	BIX01294	0.94
LSD1	S2101 (LSD1 inhibitor II)	0.95
PRMT1	AMI-1	0.85
SIRT1	SIRT1 inhibitor III	1.03
SIRT1/2	Tenovin-6	1.03
HDAC8	PCI-34051	0.92
BRD4 bromodomain	(+)-JQ1	0.84
Telomerase	TMPyP4	0.90
GLI1	Gant61	1.07
Proteasome	bortezomib	0.01
antipsychotic drug	chlorpromazine hydrochloride	1.02
depression treatment	desipramine hydrochloride	0.98

ATP content relative to that of non-treated spheroids is shown.
Effective results (<0.50) are indicated in pink.
